# Impact of body fat composition on liver iron overload severity in hemochromatosis: a retrospective MRI analysis

**DOI:** 10.1007/s11547-024-01930-8

**Published:** 2024-11-22

**Authors:** Marijan Pušeljić, Vanessa Stadlbauer, Nigar Ahmadova, Maximilian Pohl, Michaela Kopetzky, Ann-Katrin Kaufmann-Bühler, Nikolaus Watzinger, Jasminka Igrec, Michael Fuchsjäger, Emina Talakić

**Affiliations:** 1 Division of General Radiology, Department of Radiology, Medical University of Graz, Auenbruggerplatz 9, 8036 Graz, Austria; 2 Division of Gastroenterology and Hepatology, Department of Internal Medicine, Medical University of Graz, Auenbruggerplatz 15, 8036 Graz, Austria; 3 Center of Biomarker Research in Medicine (CBmed), Stiftingtalstrasse 5, 8010 Graz, Austria

**Keywords:** Hemochromatosis, Iron metabolism disorders, Body fat distribution, Magnetic resonance imaging

## Abstract

**Purpose:**

To evaluate the correlation between ectopic adipose tissue and iron overload severity in patients with hemochromatosis.

**Material and methods:**

A retrospective cohort of 52 patients who underwent liver iron concentration quantification from January 2015 to October 2023 using a 3.0T MRI scanner. R2* relaxation times and proton density fat fraction (PDFF) were assessed for the entire liver volume and a specific region of interest (ROI) placed in the right lobe. Total body fat (TF), subcutaneous fat (SCF), intermuscular fat (IMF), and visceral fat (VSF) percentages were calculated from a single axial slice at the level of the third lumbar vertebra. Additionally, ratios of IMF-to-VSF, IMF-to-SCF, and SCF-to-VSF were calculated. Standard iron laboratory parameters were collected at least one month prior to MRI. Pearson correlation coefficient was used for correlation analysis.

**Results:**

The mean age of participants was 53.9 ± 19.6 years. IMF positively correlated with R2* values in the ROI (*p* = 0.005, r_s_ = 0.382) and entire liver (*p* = 0.016, r_s_ = 0.332). Conversely, VSF negatively correlated with R2* values from the ROI (*p* = < 0.001, r_s_ = − 0.488) and entire liver (*p* = < 0.001, r_s_ = − 0.459). Positive correlations were also found between IMF-to-VSF and R2* of the ROI (*p* = 0.003, r_s_ = 0.400) and whole liver (*p* = 0.008, r_s_ = 0.364). Ferritin levels positively correlated with R2* values calculated from ROI (*p* = 0.002, r_s_ = 0.417) and whole liver volume (*p* = 0.004, r_s_ = 0.397). A positive correlation was noted between PDFF of the entire liver and TF (*p* = 0.024, rs = 0.313).

**Conclusion:**

The percentage of Intermuscular and visceral adipose tissues correlates with the severity of liver iron overload in hemochromatosis patients.

## Introduction

Magnetic resonance imaging (MRI) is the preferred method for assessing liver iron concentration (LIC) in patients suspected of iron overload. Both 1.5T and 3.0T scanners offer validated results against liver biopsy benchmarks [1, 2]. Hemochromatosis is a group of diseases characterised by total body iron excess and consecutive iron-mediated organ damage [3], which is classified as hereditary hemochromatosis (HH) or acquired hemochromatosis (AH) [4, 5]. HH involves an underlying dysregulation of one of the several iron-controlling genes which can be divided into hemochromatosis gene (HFE) related or non-HFE related hemochromatosis [4]. AH has a broad spectrum of underlying causes, ranging from hematologic disorders, repeated blood transfusion to dysmetabolic iron overload [6, 7].

Adipose tissue accumulating in areas outside of the subcutaneous layer, such as in the intermuscular or visceral compartments, is termed ectopic adipose tissue and has been recognized as an important risk factor for the development of several diseases [8–10], including their association with dysregulation of iron metabolism [7, 11, 12]. Intermuscular fat (IMF) is of particular interest, as its regulation is independent of other body fat compartments, total body weight or adiposity [13], and has an established distinct association with metabolic abnormalities [8, 12]. MRI has been commonly used to analyze fat compartments [12] and demonstrates a good level of agreement with histology [14]. A prior study with MRI-based quantification of liver iron concentration (LIC) in healthy subjects demonstrated no significant correlation with individual body fat compartments [15], while most other studies reported higher ferritin and lower serum iron values in obese patients or visceral fat without the assessment of LIC [16–18]. There is a lack of studies involving quantitative MRI based analysis of LIC and its correlation with body fat composition in metabolic disease, while its relationship in hemochromatosis remains underexplored.

The aim of this study is to evaluate the correlation between LIC and the accumulation of adipose tissue across different body fat compartments, as well as with standard liver iron parameters in patients with hemochromatosis. We hypothesize that individual body fat compartments have distinct associations with the severity of liver iron overload and show different correlations with standard liver iron parameters.

## Methods

### Study design

This retrospective monocentric study received approval from our institutional ethics committee and adhered to the principles of the Declaration of Helsinki. The need for written informed consent was waived.

Patients who underwent an MRI scan for LIC between January 2015 and October 2023 were included in this study. The inclusion criteria were as follows: (i) established diagnosis of hemochromatosis according to the clinical report of a gastroenterologist specialised in metabolic liver disease, (ii) non-invasive MRI LIC performed using the same method, and (iii) an MRI scan performed prior to any treatment, including any invasive procedure. The following exclusion criteria were defined: (i) inadequate imaging quality, (ii) incorrect liver segmentation, (iii) laboratory data sampled more than one month before the MRI scan, and (iv) the presence of focal liver lesions.

### MRI protocol

All patients underwent an MRI scan on a 3T device (MAGNETOM Skyra, Siemens Healthineers, Erlangen, Germany). LIC and fat fraction evaluation were performed using LiverLab (Siemens Healthineers, Erlangen, Germany), which includes a T1 3D volumetric interpolated breath-hold sequence (VIBE) 2-point e-Dixon and a T1 VIBE multi-echo 6-point q-Dixon. Proton density fat fractions (PDFF) and R2* relaxation times are acquired for both, the whole segmented liver volume and for a region of interest (ROI) positioned in the right lobe. For the 2-point e-Dixon 5 series are created (opposed-phase, in-phase, fat-only, water-only and water-only segmented sequence) while the 6-point q-Dixon provides 8 series (water-only, fat-only, fat percentage, Goodness-of-fit, R2* maps, T2* maps and water percentage sequence). The R2* values are corrected for fat effects and the fat percentage is corrected for the T2* effects. Goodness-of-fit provides a fit error of the liver segmentation with values over 5% indicating incorrect liver segmentation [19, 20]. Iron dry weight (IDW) was estimated using the equation: 0.314 R2* − 0.96 [1, 2] and expressed as mg Fe/g of dry weight. Full MRI protocol is shown in Table 1.

**Table 1:** Detailed scan parameters used in our MRI protocol for quantification of LIC

Variable	Sequence				
Sequence type	T2 HASTE	T2 HASTE	T2 HASTE FS	VIBE e-Dixon	VIBE q-Dixon
Scan mode	2D	2D	2D	3D	3D
Acquisition plane	Axial	Coronal	Axial	Axial	Axial
Repetition time (ms)	1600	1080	1200	3.92	9.55
Number of echoes (n)	1	1	1	2	6
Initial echo time (ms)	95	87	95	1.24	1.43
Flip angle (°)	160	90	156	9	9
Slice thickness (mm)	5	5	5	3.5	3
Field of view (mm^3^)	308 × 380	400 × 400	300 × 500	393 × 450	402 × 460
Percent phase field of view (%)	81.25	100	81.25	81.25	84.375
Pixel Bandwidth	710	700	710	1020	1040
Acquisition matrix	320 × 208	256 × 256	320 × 208	288 × 175	320 × 203
Echo train length	87	100	87	2	6

*HASTE* Half fourier single-shot turbo spin-echo, *FS* Fat suppression, *VIBE* Volumetric interpolated breath-hold sequence

### MRI analysis

Two radiologists (MP, ET) performed MR image analysis. Interpretative differences were resolved through consensus. Measurements included the liver's maximal anteroposterior (AP), mediolateral (ML), and craniocaudal (CC) dimensions [21]. Based on qualitative visual assessment, the liver surface was categorized as smooth, slightly irregular, or nodular, and the parenchymal signal structure as either homogeneous or heterogeneous. Visual comparisons of opposed-phase and in-phase sequences were made to identify liver steatosis (lower signal intensity in the opposed phase) and iron overload (lower signal intensity in the in-phase) [22, 23].

### Fat compartment analysis

**Fig. 1:**
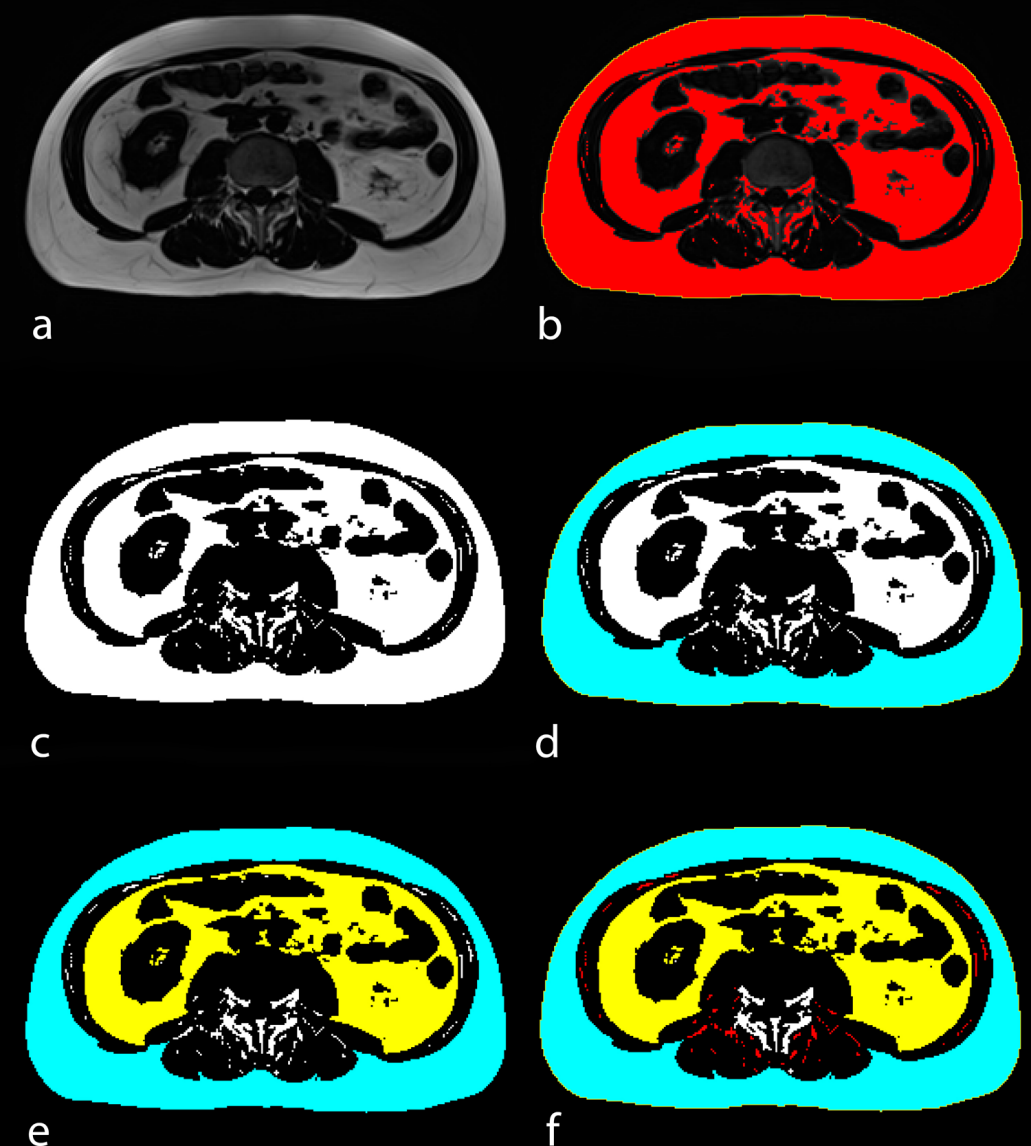
Example of a body fat compartment segmentation. **a** an axial slice at the level of the third lumbar vertebra is selected. **b** A threshold is selected covering most of the visible fat tissue (marked red). Area measurement of the whole slice is performed without limiting the measurement to the applied threshold. **c** After applying the threshold, fat tissue is colored white. After segmenting SCF (**d**, colored blue), VSF (**e**, colored yellow) and IMF (**f**, colored red), the area of each compartment is calculated. *SCF* subcutaneous fat, *VSF* Visceral fat, *IMF* Intermuscular fat

The analysis of fat compartments was conducted by two radiologists (MP, AKB) using the open-source software ImageJ (version 1.54 g, U.S. National Institutes of Health, Bethesda, MD, USA). From each MRI exam, a single axial slice from a fat-only T1 VIBE Dixon sequence, which exhibited high contrast between fat tissue and other adjacent structures at the level of the third lumbar vertebra, was selected for analysis. The methodology was adapted from Gomez-Perez et al. [24], with adjustments for MRI-specific thresholds. Figure 1 provides a more detailed example of our performed fat compartment segmentation.

The total area of the selected single axial slice and the areas for subcutaneous fat (SCF), visceral fat (VCF), and intermuscular fat (IMF) were measured in mm^2^, with total fat area derived and expressed as percentages. The percentage of Total body fat (TF) was derived from the entire slice area, while percentages for each fat compartment were calculated as a proportion of the TF. Additionally, we determined the intermuscular-to-visceral fat (IMF-to-VSF), intermuscular-to-subcutaneous fat (IMF-to-SCF), and subcutaneous-to-visceral fat (SCF-to-VSF) ratios.

### Clinical and laboratory data

Clinical and laboratory data were obtained retrospectively from the hospital’s information system. Demographic data (age at diagnosis, sex), current smoking status and alcohol consumption, body mass index (BMI), hemochromatosis type, presence of selected chronic diseases (arterial hypertension, diabetes mellitus, coronary heart disease, dyslipidemia, chronic obstructive lung disease), iron parameters (serum iron, ferritin, transferrin, transferrin saturation), liver and renal function parameters were gathered.

### Statistical analysis

Statistical analysis was performed using SPSS v.29.0 software (SPSS Inc., Chicago, IL, USA). A *P*-value < 0.05 was considered statistically significant. Pearson correlation coefficient was used for correlation analysis, based on the data distribution and outliers, which were tested both visually, using QQ-plots, and statistically, with Kolmogorov–Smirnov test. Correlation was assessed both for the entire study sample and individual groups (HH and AH). Continuous variables are expressed as mean ± standard deviation, along with their ranges, while categorical variables are presented as numbers, proportions, and percentages. Continuous variables with a normal distribution were compared using the Student’s t-test, whereas the Mann–Whitney U test was used for independent samples in the absence of normality, and the Wilcoxon signed-rank test was used for paired comparisons. Pearson’s chi-squared and Fisher’s exact tests were used for the comparison of categorical variables. After applying a log10 transformation to normalize skewed data, multiple linear regression was conducted to assess the impact of the group variable (hemochromatosis type) on the relationship between LIC, standard iron laboratory parameters and body fat compartment measurements. Two separate models were created for each fat compartment and laboratory parameter: one using R2* values from the ROI and another using R2* values from the whole segmented liver.

## Results

### Study group characteristics

**Fig. 2:**
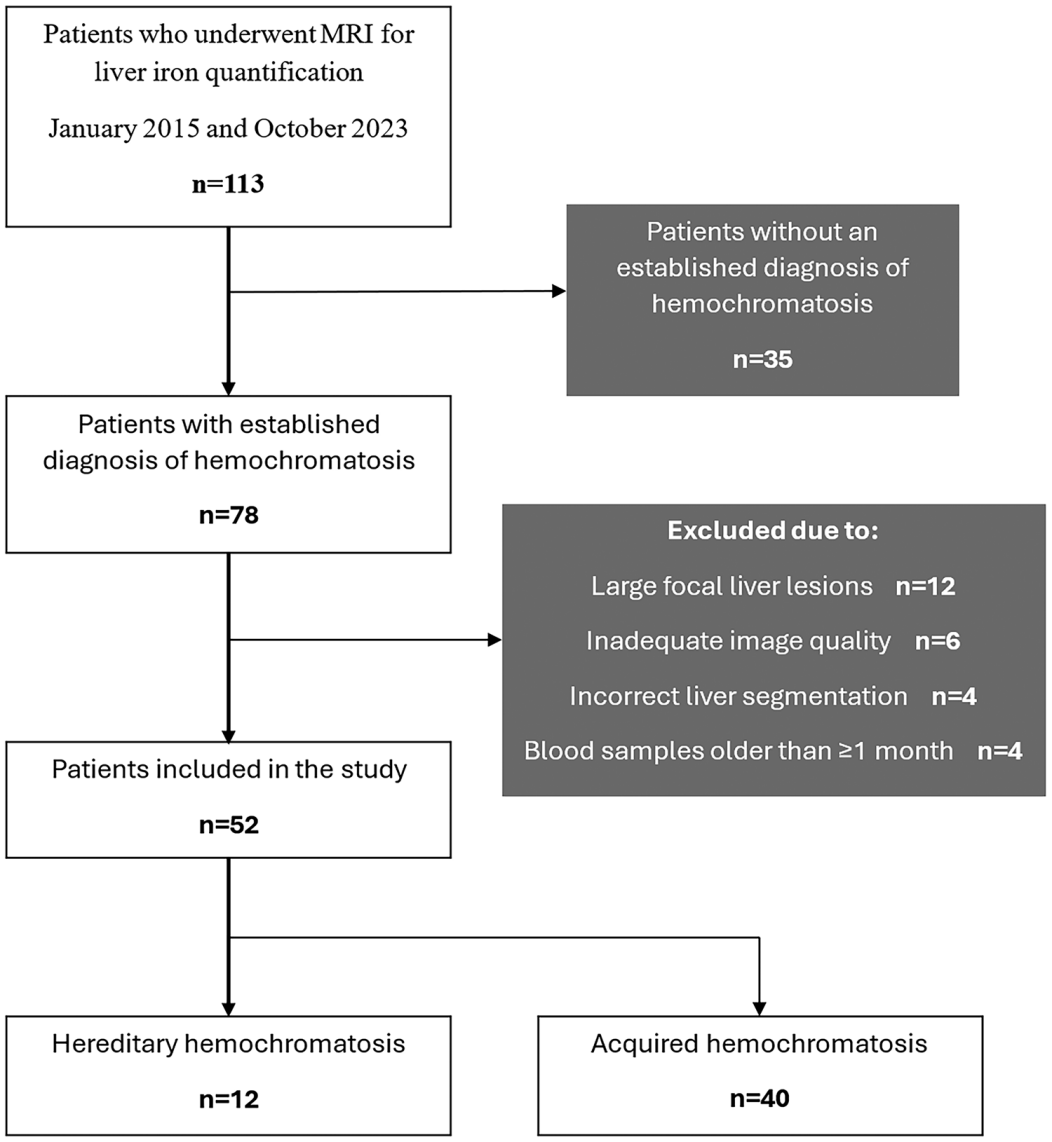
Flowchart of this study

A total of 52 patients were included in this study (Fig. 2) with a mean age of 53.9 ± 19.6 [SD] years (range 7–83 years), comprising 41 males (78.8%) and 11 females (21.2%). Out of these patients, 12 (23.1%) had hereditary hemochromatosis (HH), while the remaining 40 (76.9%) were diagnosed with acquired hemochromatosis (AH). The most common underlying condition for AH was dysmetabolic iron overload syndrome (DIOS, n = 30, 75.0%), followed by myelodysplastic syndromes (n = 5, 12.5%), repeated blood transfusions (n = 4, 10.0%), and hemoglobinopathy (n = 1, 2.5%). All patients with HH had a type 1 HH (HFE-related) (4). Baseline characteristics and group comparison are shown in Table 2.

**Table 2:** Baseline characteristics of the study population

Parameter	Whole sample (n = 52)	Hereditary Hemochromatosis (n = 12)	Acquired Hemochromatosis (n = 40)	Group comparison
Age (years)	53.9 ± 19.6 (7–83)	49.1 ± 20.8 (9–78)	55.3 ± 19.3 (7–83)	*p* = 0.273
Sex (male:female)	41:11 (78.85%:21.15%)	10:2 (83.3%:16.7%)	31:9 (77.5%:22.5%)	*p* = 0.720
BMI (kg/m^2^)	26.0 ± 5.0 (12.3–37.9)	26.8 ± 6.6 (12.3–37.9)	25.8 ± 4.6 (16.3–35.3)	*p* = 0.254
Current smoker, No. (%)	7/52 (13.5%)	2/12 (16.7%)	8/40 (20.0%)	*p* = 0.714
Alcohol consumers, No. (%)	18/52 (34.6%)	4/12 (33.3%)	14/40 (35.0%)	*p* = 0.601
Arterial hypertension, No. (%)	18/52 (34.6%)	6/12 (50%)	12/40 (30.0%)	*p* = 0.175
Diabetes mellitus, No. (%)	5/52 (9.6%)	1/12 (8.3%)	4/40 (10.0%)	*p* = 0.675
Coronary heart disease, No. (%)	6/52 (11.5%)	1/12 (8.3%)	5/40 (12.5%)	*p* = 0.576
Dyslipidemia, No. (%)	19/52 (36.5)	6/12 (50.0%)	13/40 (32.5)	*p* = 0.221
COPD, No. (%)	0/52 (0%)	0/12 (0%)	0/40 (0%)	Not applicable
Hemoglobin (g/dL)	13.8 ± 2.7 (5.7–18.2)	14.0 ± 2.5 (10.10–17.40)	13.7 ± 2.8 (5.7–18.2)	*p* = 0.542
Erytrhocytes (10^12^/L)	4.3 ± 0.9 (1.9–5.8)	4.4 ± 0.9 (2.9–5.5	4.3 ± 0.9 (1.9–5.8)	*p* = 0.582
Hemoatocrit (%)	39.7 ± 7.6 (15.9–51.5)	40.9 ± 7.0 (29.0–48.2)	39.3 ± 7.9 (15.9–51.5)	*p* = 0.433
eGFR (mL/min)	82.7 ± 25.8 (13.3–157.0)	82.3 ± 41.2 (13.3–157.0)	82.8 ± 20.1 (34.7–116.3)	*p* = 0.566
Iron (μg/dL)	160.5 ± 89.4 (29 – 629)	161.0 ± 61.3 (83–297)	160.4 ± 96.6 (29–629)	*p* = 0.656
Ferritin (ng/mL)	1526.5 ± 2371.6 (43–16,470)	901.8 ± 542.1 (182–2108)	1718.6 ± 2674.8(43–16,470)	*p* = 0.253
Transferrin (g/L)	2.1 ± 0.6 (0.8–3.4)	1.9 ± 0.7(0.8–3.1)	2.2 ± 0.6 (0.8–3.4)	*p* = 0.386
Transferrin saturation (%)	52.3 ± 22.0 (16 – 97)	60.7 ± 20.5 (32.00–96.31)	49.7 ± 22.1 (16–97)	*p* = 0.076

*BMI* Body mass index, *COPD* Chronic obstructive lung disease, *eGFR* Estimated glomerular filtration rate

### Body fat composition and LIC

There was no statistically significant difference between the acquired R2* measurements from the ROI (172.9 ± 142.8 s⁻^1^ [SD], range 13.6–788.4 s⁻^1^) and from the whole liver volume (155.8 ± 92.3 s⁻^1^ [SD], range 31.3–484.9 s⁻^1^; *p* = 0.560).

**Fig. 3:**
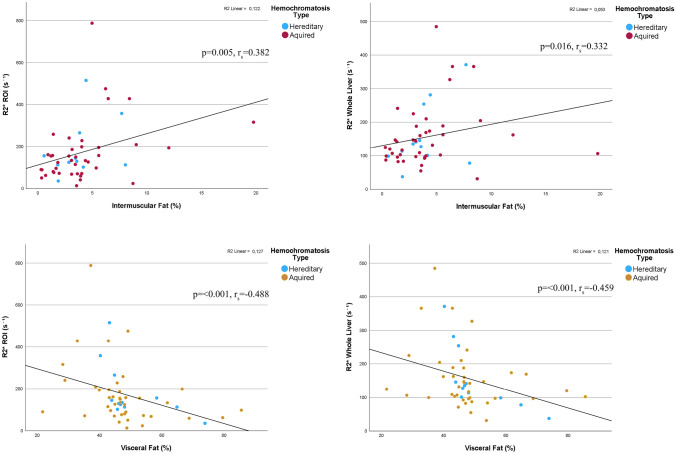
Correlation between IMF and VSF to R2* values calculated from a ROI placed in the right lobe and form the whole segmented liver volume. *IMF* Intermuscular far, *VSF* Visceral fat, *ROI* Region of interest

**Fig. 4:**
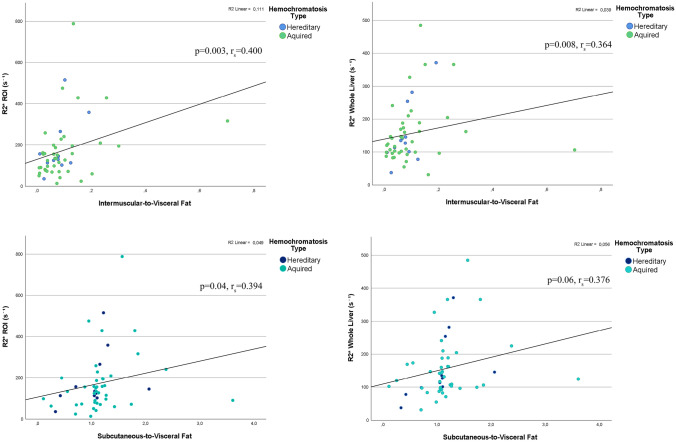
Correlation between ratios of IMF-to-VSF and SCF-to-IMF to R2* values calculated from a ROI placed in the right lobe and form the whole segmented liver volume. *IMF* Intermuscular far, *VSF* Visceral fat, *ROI* Region of interest

IMF showed a positive correlation with R2* values measured in the ROI (*p* = 0.005, r_s_ = 0.382) and for the entire segmented liver volume (*p* = 0.016, r_s_ = 0.332) (Fig. 3). Conversely, VSF showed a negative correlation with R2* values from the ROI (*p* = < 0.001, r_s_ = − 0.488) and from the entire segmented liver volume (*p* = < 0.001, r_s_ = − 0.459). Additionally, a positive correlation was observed between IMF-to-VSF ratio and R2* of the ROI (*p* = 0.003, r_s_ = 0.400) and the whole segmented liver (*p* = 0.008, r_s_ = 0.364) (Fig. 4). SCF-to-VSF ratio exhibited a similar positive correlation with R2* values measured in the ROI (*p* = 0.04, r_s_ = 0.394) and the whole liver (*p* = 0.06, r_s_ = 0.376).

HH group had a stronger negative correlation between VSF and R2* values from the ROI (*p* = 0.011, r_s_ = − 0.699) and whole liver (*p* < 0.001, r_s_ = − 0.895) in comparison to AH group (ROI *p* = 0.003, r_s_ = − 0.455, whole liver *p* = 0.024, r_s_ = − 0.357). AH group had statistically significant correlation between IMF and R2* values from the ROI (*p* = 0.013, r_s_ = 0.388) and whole liver (*p* = 0.038, r_s_ = 0.330), while HH showed only a non-statistically significant positive correlation (ROI *p* = 0.471, r_s_ = 0.231, whole liver *p* = 0.217, r_s_ = 0.385). Complete results are shown in Tables 3 and 4.

**Table 3:** Body fat distribution percentages, R2* relaxation times and estimated LIC of the study population

Parameter	Whole study sample (n = 52)	Hereditary Hemochromatosis (n = 12)	Acquired Hemochromatosis (n = 40)	Group comparison
Total fat (%)	55.6 ± 17.6 (21.3–90.5)	53.4 ± 18.7 (24.4–90.2)	56.3 ± 17.4 (21.3–90.5)	*p* = 0.811
Subcutaneous fat, % of Total fat	48.6 ± 12.8 (9.2–90.2)	49.2 ± 16.2 (5.8–63.7)	48.4 ± 11.8 (9.2–78.1)	*p* = 0.645
Visceral fat, % of Total fat	48.1 ± 11.7 (21.7–85.5)	50.2 ± 10.1 (40.2–73.8)	47.5 ± 12.2 (21.7–85.5)	*p* = 0.676
Intermuscular fat, % of Total fat	4.0 ± 3.3 (0.3–19.8)	3.8 ± 2.2 (0.6–8.0)	4.1 ± 3.6 (0.3–19.8)	*p* = 0.957
R2* values for ROI (s⁻^1^)	172.9 ± 142.8 (13.6–788.4)	183.0 ± 133.7(36.0–515.7)	169.8 ± 146.9(13.6–484.9)	*p* = 0.617
R2* values for whole liver (s⁻^1^)	155.8 ± 92.3 (31.3–484.9)	157.0 ± 92.0(37.5–371.2)	155.5 ± 92.4(31.3–484.9)	*p* = 0.983
Iron dry weight from ROI (mg/g)	3.0 ± 2.5 (0.2–13.8)	3.2 ± 2.4(0.6–9.0)	2.9 ± 2.6(0.2–13.8)	*p* = 0.617
Iron dry weight from whole liver (mg/g)	2.7 ± 1.6 (0.5–8.5)	2.7 ± 1.7(0.6–6.5)	2.7 ± 1.6(0.5–8.5)	*p* = 0.983

*LIC* Liver iron concentration, *ROI* Region of interest

**Table 4:** Results of the performed spearman correlation analysis between PDFF, R2* values, body fat composition and standard iron laboratory parameters

Parameter	Iron	Ferritin	Transferrin	Transferrin saturation
R2* ROI	*p* = 0.573, r_s_ = 0.082	*p* = 0.002*, r_**s**_** = 0.417**	*p* = 0.111, r_s_ = − 0.228	*p* = 0.717, r_s_ = 0.052
R2* Whole liver	*p* = 0.535, r_s_ = 0.090	*p* = 0.004*, r_**s**_** = 0.397**	*p* = 0.336, r_s_ = − 0.139	*p* = 0.882, r_s_ = − 0.021
PDFF ROI	*p* = 0.013*, r_**s**_** = 0.351**	*p* = 0.286, r_s_ = 0.152	*p* = 0.021*, r_**s**_** = 0.325**	*p* = 0.515, r_s_ = 0.093
PDFF Whole liver	*p* = 0.018*, r_**s**_** = 0.333**	*p* = 0.803, r_s_ = 0.036	*p* = 0.021*, r_**s**_** = 0.325**	*p* = 0.559, r_s_ = 0.084
BMI	*p* = 0.792, r_s_ = − 0.038	*p* = 0.553, r_s_ = 0.085	*p* = 0.415, r_s_ = − 0.118	*p* = 0.343, r_s_ = − 0.135
Total Fat	*p* = 0.846, r_s_ = − 0.028	*p* = 0.161, r_s_ = − 0.199	*p* < 0.001*, r_**s**_** = 0.457**	*p* = 0.104, r_s_ = − 0.230
Subcutaneous Fat	*p* = 0.153, r_s_ = 0.205	*p* = 0.086, r_s_ = 0.243	*p* = 0.981, r_s_ = − 0.003	*p* = 0.722, r_s_ = 0.051
Visceral Fat	*p* = 0.107, r_s_ = − 0.231	*p* = 0.012*, r_**s**_** = **− **0.349**	*p* = 0.935, r_s_ = − 0.012	*p* = 0.598, r_s_ = − 0.076
Intermuscular Fat	*p* = 0.964, r_s_ = 0.007	*p* = 0.302, r_s_ = 0.147	*p* = 0.738, r_s_ = 0.049	*p* = 0.974, r_s_ = 0.005

*ROI* Region of interest, *PDFF* Proton density fat fraction, *BMI* Body mass index

*Statistically significant

Bolded p-values indicate statistical significance, defined as *p*<0.05

### *Iron* laboratory parameters

A negative correlation was observed between VSF and ferritin values (*p* = 0.012, r_s_ = − 0.349), while a positive correlation existed between transferrin values and TF (*p* < 0.001, r_s_ = 0.457). Ferritin showed a positive correlation with R2* values calculated from ROI (*p* = 0.002, r_s_ = 0.417) and the entire liver volume (*p* = 0.004, r_s_ = 0.397). No significant correlation was observed between R2* values and serum iron, transferrin, or transferrin saturation. Complete results are shown in Tables 3 and 4.

### Impact of hemochromatosis type

In multiple linear regression after adjustment for confounding factor (hemochromatosis type) the associations were still significant between R2* ROI and VSF (β = − 0.39, 95% CI − 0.21 to − 0.04, *p* = 0.005) and IMF (β = 0.32, 95% CI 0.05 to 0.69, *p* = 0.024), whereas the association to SCF was without statistical significance (β = 0.27, 95% CI − 0.01 to 0.25, *p* = 0.052). Similarly, the association between R2* from the whole liver and SCF (β = 0.30, 95% CI 0.02 to 0.37, *p* = 0.031) and VSF remained significant (β = − 0.37, 95% CI − 0.28 to − 0.04, *p* = 0.008), while the association with IMF lost its statistical significance (β = − 0.26, 95% CI − 0.03 to 0.88, *p* = 0.064).

The association between VSF and ferritin remained statistically significant (β = − 0.35, 95% CI − 0.14 to 0.02, *p* = 0.012), same as between TF and transferrin (β = 0.48, 95% CI 26.57 to 15.78, *p* < 0.001). Additionally, the association between ferritin and R2* ROI (β = 0.59, 95% CI 0.48 to 1.10, *p* < 0.001) and R2* form the whole liver (β = 0.52, 95% CI 0.51 to 1.43, *p* < 0.001) remained statistically significant.

### Liver steatosis

By visual assessment, liver steatosis was present in 32.07% (n = 17) of the patients, with no significant difference between the HH (33.33%, n = 4) and AH (32.50%, n = 13, *p* = 0.608). Lower signal intensity in the in-phase sequence was present in 12 patients (23.10%), with no difference between HH (33.33%, n = 4) and AH (20.00%, n = 8, *p* = 0.437). A statistically significant difference was observed between the PDFF measurements from the ROI (11.6 ± 10.7% [SD], range 0.2–37.3%) and the whole liver volume (15.4 ± 10.9% [SD], range 2.1–38.9%; *p* < 0.001).

A positive correlation was noted between the PDFF of the entire liver and TF (*p* = 0.024, r_s_ = 0.313) and between BMI and PDFF from the ROI (*p* = 0.022, r_s_ = 0.331). There was no statistically significant correlation between R2* values from the ROI and PDFF measured in the ROI (*p* = 0.580, r_s_ = 0.079) or the entire liver volume (*p* = 0.775, r_s_ = 0.041). Correlation between PDFF and laboratory iron parameters is shown in Table 4.

### Liver morphology

The ML liver diameter showed a positive correlation with VSF (*p* = 0.031, r_s_ = 0.300), TF (*p* < 0.001, r_s_ = 451), BMI (*p* < 0.001, r_s_ = 0.649), and a negative correlation with the SCF-to-VSF ratio (*p* = 0.024, r_s_ = − 0.313). BMI also showed a positive correlation with AP (*p* = 0.037, r_s_ = 0.303) and CC (*p* = 0.045, r_s_ = 0.291) liver sizes. R2* values from the ROI had a negative correlation with liver AP (*p* = 0.022, r_s_ = − 0.316), ML (*p* = 0.043, r_s_ = − 0.281), and CC diameters (*p* = 0.014, r_s_ = − 0.339). Similar results were found for R2* values estimated from the whole liver regarding AP (*p* = 0.011, r_s_ = − 0.351), ML (*p* = 0.071, r_s_ = − 0.253), and CC liver diameter (*p* = 0.005, r_s_ = − 0.339).

The liver surface was graded as smooth in 47 (90.4%) patients, slightly irregular in 4 patients (7.7%) and nodular in 1 patient (1.9%). The liver parenchyma was homogenous in 32 patients (61.5%), moderately inhomogeneous in 18 patients (34.6%), and strongly inhomogeneous in 2 patients (3.8%). There were no significant differences between groups regarding liver surface (*p* = 0.074) and parenchyma homogeneity (*p* = 0.329). Furthermore, there was no statistical significance regarding liver surface and R2* values (ROI *p* = 0.883, whole liver *p* = 0.356). Additionally, no statistical significance was observed for parenchymal homogeneity and R2* values (ROI *p* = 0.802, whole liver *p* = 0.584).

## Discussion

This is the first study that performed a quantitative MRI-based LIC and body fat compartment analysis in patients with hemochromatosis. Our findings indicate that it is not the overall adipose accumulation that correlates with iron overload severity but rather the distribution of fat across different body compartments that shows a significant relationship with the degree of liver iron overload. Specifically, a higher percentage of IMF was associated with more severe iron overload, while a higher percentage of VSF had the opposite correlation and was associated with lower liver iron overload. Moreover, an elevated IMF-to-VSF ratio correlated with more pronounced liver iron overload. Additionally, while a higher BMI was associated with increased PDFF, it was not associated with the severity of LIC.

We performed quantification of liver iron using R2* relaxometry, which is a well-validated first line method for noninvasive evaluation of LIC [25]. No statistically significant difference was observed between the results obtained from the ROI placed in the right lobe and from the whole segmented liver volume. A heterogeneous iron distribution is common among healthy individuals, while in patients with moderate to high liver iron overload, the variability of iron deposition between different liver segments is either insignificant [25] or small [26]. Our results reflect a potentially more homogeneous distribution of iron deposition in hemochromatosis. In contrast, we observed a statistically significant difference between the PDFF results obtained from the ROI and from the whole segmented liver volume, which aligns with the reported high variability of fat fractions, up to 5%, between individual segments [26].

Both patients with HH and AH were included in our study, each having a different underlying mechanism for liver iron overload, with hepcidin being the main regulatory molecules for iron metabolism affected in both conditions [27–29]. In HH, a molecular defect leads to deficient synthesis or reduced activity of hepcidin, resulting in uncontrolled intestinal iron absorption and subsequent accumulation in the liver parenchyma [4, 28]. Some forms of AH, such as those associated with myelodysplastic syndrome, hemoglobinopathies, and repeated blood transfusions, are caused by a down-regulation of hepcidin mediated through anemia- and hypoxia-associated signaling pathways, leading to a similar effect as seen in HH with increased intestinal iron absorption [6, 30]. In DIOS, a common form of AH, elevated hepcidin concentrations are observed in hepatocytes and macrophages, leading to cellular retention of iron [6]. Additionally, other mechanisms, such as hepcidin resistance with increased intestinal absorption of iron, have also been proposed for DIOS [29]. Although AH is commonly associated with milder liver iron overload in comparison to HH [32], some forms of AH can have significant levels of LIC [6, 30]. In our study, no statistically significant difference was observed between the acquired results for R2* values from the ROI and from the whole segmented liver volume between AH and HH, even though the numerical imbalance between the groups needs to be taken into consideration.

The relationship of obesity and adipose tissue accumulation with iron metabolism have been thoroughly evaluation. Obesity, especially the VSF compartment, has been associated with a low-grade chronic inflammatory state due to the activation of the non-specific immune system, which increases the production of pro-inflammatory cytokines such as tumor necrosis factor-alpha and Interleukin-6 [7, 31, 32]. Hepcidin is affected by this proinflammatory state, leading to overexpression of it with consecutive intracellular retention of iron, increased body iron stores, elevated serum ferritin, and a decrease in serum iron and transferrin saturation [29, 32]. Previous studies have demonstrated a positive correlation between ferritin levels and VSF, SCF, and BMI [11, 18, 33]. Additionally, a correlation has been observed between central adiposity and higher hepcidin values, along with lower transferrin saturation [17, 34]. Regardless of the fat compartment analyzed, obese patients tend to exhibit a high prevalence of liver iron overload and elevated serum ferritin levels [35]. Although most of these studies focus on healthy subject or patients with AH, similar observation have also been reported for HH, where confounding factors such as metabolic syndrome and obesity predispose patients to iron overload more than the genetic component itself [6, 36, 37].

In contrast, some studies have established an opposite correlation between obesity and iron overload, such as non-expression phenotypes of HH associated with obesity [15], or a protective role of hemochromatosis against obesity in both humans [38] and mice [39]. Interestingly, therapeutic measures for reducing iron overload, such as phlebotomy, are followed by weight gain [40, 41]. The underlying mechanism for this protective effect of obesity could be the overexpression of hepcidin in adipose tissue, while its expression in hepatocytes remains unchanged, leading to iron accumulation in adipose tissue rather than in the liver parenchyma [15]. An alternative explanation could be iron-mediated inhibition of lipogenesis and lipidosis, where patients with HH may exhibit less adipose tissue [7, 38, 39]. Furthermore, adipose tissue appears to be more sensitive to iron accumulation than liver parenchyma when iron overload occurs more rapidly [42].

In our study, higher percentages of VSF were associated with a less severe liver iron overload. This finding aligns more closely with studies that reported a protective role of adiposity in hemochromatosis [7, 39, 42]. Unlike previous findings, our study showed a negative correlation between VSF and serum ferritin values, while no significant correlation was found between BMI, body fat compartments, or ferritin values. Furthermore, although previous studies reported a negative correlation between transferrin saturation and both BMI and central obesity [43], such correlations were not observed in our study. This difference in results could be explained by the different study populations.

IMF has been explored in a variety of diseases [8–10] and, similar to VSF, has also been associated with the production of pro-inflammatory cytokines and chronic inflammatory state [44, 45]. Moreover, some studies suggest that IMF may have a greater metabolic impact compared to SCF or VSF [10], with regulatory mechanism and inflammatory cascade independent of other body fat compartments [13, 32]. However, no comparable studies are focusing on IMF in relation to liver iron overload or MRI-based LIC quantification. In our study, we observed that patients with higher LIC had a higher percentage of IMF. Similar to other studies involving IMF, the precise mechanism remains unclear. The proinflammatory effects of IMF could be a potential underlying cause, though alternative explanations cannot be dismissed due to the scarcity of studies on IMF in patients with hemochromatosis.

Our study, despite its retrospective design, faces several limitations. Firstly, we included patients with AH and HH, which are conditions with different underlying etiologies that both influence the function of hepcidin [7, 34, 46]. Secondly, our study sample is relatively small and inhomogeneous with an imbalance between the two groups, which may limit our statistical power, especially in the detection of some differences between AH and HH. Even though we performed a linear regression analysis for the impact of hemochromatosis type on the results, the effect of the group imbalance can not be ruled out. Additionally, our study exclusively involved patients with HFE-related HH, characterized by low penetrance and significantly influenced by host-related or environmental cofactors (4). Thirdly, the majority of our study sample was male, partially reflecting the higher prevalence of some HH forms in males [47]. However, the severity of liver iron overload has been found to be similar in both men and women [48]. Lastly, the estimation of percentages of body fat distribution was based on a two-dimensional single axial slice, which may not accurately represent the actual body fat distribution in individual patients. However, the measurement of body fat at the level of the second or third lumbar vertebra has been shown to sufficiently predict total body fat volume [49].

## Conclusion

Our research suggests a complex interplay between iron and fat metabolism in individuals with hemochromatosis. The amount of adipose tissue in the intermuscular and visceral compartment appears to have an influence on the severity of iron overload, while this was not observed for the subcutaneous fat compartment. The interaction between anti-inflammatory and pro-inflammatory pathways, as well as iron-mediated effects on adipose tissue, may serve as connecting mechanisms between liver parenchyma and fat compartments. Further research is essential to understand the therapeutic implications and prognostic stratification of patients with hemochromatosis based on their body fat distribution.
